# The Physician’s Visiting List for 1885

**Published:** 1884-12

**Authors:** 


**The Physician's Visiting List** (Lindsay & Blakiston) for 1885. Philadelphia: P. Blakiston, Son & Co., 1012 Walnut street.

With the approach of '85 the usual number of physicians' visiting lists are offered to the profession. The majority, however, hold to this old favorite, which, for thirty-four years, has appeared and which seems to flourish upon the competition. In our own opinion there is none better.

